# One-pass deep brain stimulation of dentato-rubro-thalamic tract and subthalamic nucleus for tremor-dominant or equivalent type Parkinson’s disease

**DOI:** 10.1007/s00701-016-2725-4

**Published:** 2016-02-15

**Authors:** Volker Arnd Coenen, Michel Rijntjes, Thomas Prokop, Tobias Piroth, Florian Amtage, Horst Urbach, Peter Christoph Reinacher

**Affiliations:** Department of Stereotactic and Functional Neurosurgery, Freiburg University Medical Center, Freiburg, Germany; Department of Neurology, Freiburg University Medical Center, Freiburg, Germany; Department of Neuroradiology, Freiburg University Medical Center, Freiburg, Germany

**Keywords:** Deep brain stimulation, Dentato-rubro-thalamic tract, Fiber tracking, STN, Parkinson’s disease, Tremor

## Abstract

**Background:**

Refractory tremor in tremor-dominant (TD) or equivalent-type (EQT) idiopathic Parkinson’s syndrome (IPS) poses the challenge of choosing the best target region to for deep brain stimulation (DBS). While the subthalamic nucleus is typically chosen in younger patients as the target for dopamine-responsive motor symptoms, it is more complicated if tremor does not (fully) respond under trial conditions. In this report, we present the first results from simultaneous bilateral DBS of the DRT (dentato-rubro-thalamic tract) and the subthalamic nucleus (STN) in two elderly patients with EQT and TD IPS and dopamine-refractory tremor.

**Methods:**

Two patients received bilateral octopolar DBS electrodes in the STN additionally traversing the DRT region. Achieved electrode positions were determined with helical CT, overlaid onto DTI tractography data, and compared with clinical data of stimulation response.

**Results:**

Both patients showed immediate and sustained improvement of their tremor, bilaterally.

**Conclusions:**

The proposed approach appears to be safe and feasible and a combined stimulation of the two target regions was performed tailored to the patients’ symptoms. Clinically, no neuropsychiatric effects were seen. Our pilot data suggest a viable therapeutic option to treat the subgroup of TD and EQT IPS and with tremor as the predominant symptom. A clinical study to further investigate this approach (OPINION: www.clinicaltrials.gov; NCT02288468) is the focus of our ongoing research.

## Introduction

Tremor is the most salient motor symptom of Parkinson’s disease (IPS = idiopathic Parkinson syndrome) and other symptoms are bradykinesia, rigidity, and postural instability. Up to 75 % of patients show resting tremor [16]. Initially, tremor is typically unilateral and only visible in stressful situations. In the later stage of the disease, it becomes bilateral. Patients might in later stages show a postural and/or kinetic tremor as well, usually with the same tremor frequency [8]. Besides fluctuations, therapy refractory tremor is one of the main indications of DBS [12], which has become a standard treatment for the advanced stages [9, 25].

First studies have shown that thalamic DBS, which targets the ventral intermediate nucleus of thalamus (Vim), can effectively (95 %) reduce tremor in IPS. In larger cohorts, this number was somewhat reduced to 85 % favorable outcome [17, 20]. We have recently provided evidence that a fiber structure (DRT = dentate-rubro-thalamic tract) that traverses the thalamic (Vim) region might be the true target of thalamic DBS. This structure can directly be targeted with special tractography DTI MRI (magnetic resonance imaging) sequences [3] and allows for a perturbation of the tremor-reducing network also in the subthalamic region.

While tremor can be effectively controlled over years, bradykinesia and rigidity might not be well controlled under thalamic DBS and additional medication [20]. In turn the use of subthalamic nucleus (STN) DBS in a typically younger population shows effects on the other symptoms of IPS [23] but has no such dramatic and instantaneous tremor reducing effects as thalamic DBS [14], especially if tremor is not a dopamine-responsive symptom. There are anecdotal reports on the inferiority of pure STN DBS for tremor reduction with the need to additionally stimulate the thalamic region. Publications on this issue are lacking, however almost every group treating movement disorders knows such patients. Although older patients who suffer from tremor-dominant IPS are less likely to develop motor fluctuations, they might at a later stage of the disease suffer from insufficient symptom control of the other dopaminergic symptoms and these patients would potentially benefit from additional subthalamic surgery [11, 19]. However, because of the higher neuropsychiatric complication rate due to stimulation of the STN in this patient group [25], STN DBS is typically not recommended [11, 19].

**Fig. 1:**
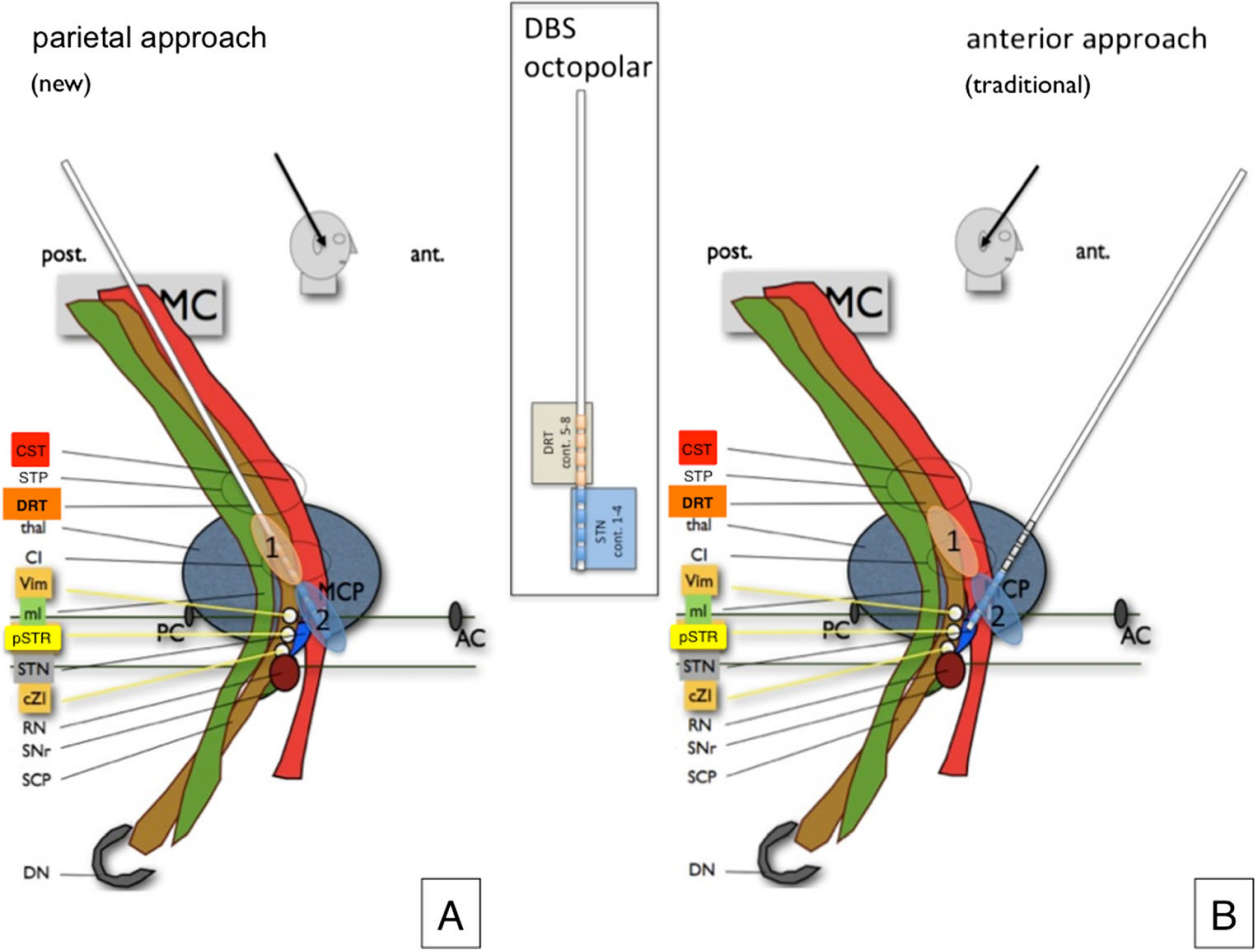
Graphic depiction of the DRT topography. The newly proposed (**a**) approach and the traditional approach (**b**) to the subthalamic nucleus (STN) with DRT (1) and STN (2) stimulation sites. *Inset* concept of the use of the octopolar electrode with STN stimulation at the tip (contacts 1–4) and DRT stimulation at the more proximal contacts (5–8). *AC* anterior commissure, *PC* posterior commissure, *MCP* mid-commissural point, *MC* primary motor cortex, m1, *CST* cortico-spinal tract, *STP* superior thalamic peduncle, *DRT* dentato-rubro-thalamic tract, *thal* thalamus, *CI* internal capsule, *Vim* ventral intermediate nucleus of thalamus stereotactic target (possibly this is the *Vop* ventralis oralis posterior nucleus), *ml* medial lemniscus, *pSTR* posterior subthalamic region, *STN* subthalamic nucleus, *cZI* caudal zona incerta, *RN* red nucleus, *SNr* substantia nigra, *SCP* superior cerebellar peduncle, *DN* entate nucleus

Taking these points into account, an interesting consideration might be an earlier and one-pass thalamic and subthalamic implantation of DBS electrodes and a combined and tailored deep brain stimulation strategy for both target regions (STN and DRT), which can be adjusted according to the individual patient’s symptoms over the course of the disease (cf. Fig. 1). The presented work aims at the presentation of this concept for TD IPS or patients with EQT IPS who perceive tremor to be their dominant symptom.

## Materials and methods

We report on two cases of patients with IPS who had tremor as their predominant symptom. They were determined as therapy refractory and selected according to consensus guidelines as reported in the literature for DBS surgery [9, 11, 12].

### Ethics

All procedures performed in studies involving human participants were in accordance with the ethical standards of the institutional and/or national research committee and with the 1964 Helsinki Declaration and its later amendments or comparable ethical standards. Informed consent was obtained from all individual participants included in the study. Since the trajectory that is used to reach the STN is not part of the approval process (CE mark) for the DBS device, the approach is left to the discretion of the surgeon. In order to allow for a co-stimulation of two tremor targets (STN and DRT), we chose for a parietal image-assisted approach. The combined stimulation can be regarded as “compassionate use”. The presentation of DTI fiber tractographic results together with clinical results received Freiburg University institutional review board approval (No. 567/14).

### Imaging

Anatomical and diffusion tensor imaging was performed on a clinical 3-Tesla MRI system (Siemens Magnetom Trio Tim System 3T, Erlangen, Germany) a day before surgery under mild sedation with oral Lorazepam (1–2.5 mg, Pfizer, Berlin, Germany). Anatomical sequences: 12-channel head coil, 3D MPRAGE (Magnetisation Prepared Rapid Gradient Echo): TR 1 390 ms, TE 2.15 ms, TI 800 ms, flip angle 15°, voxel size 1.0 × 1.0 × 1.0 mm^3^, acquisition time 3:15. 3D T2 SPACE-sequence: TR 2 500 ms, TE 231 ms, echo train length 141, flip angle variable, voxel size 1.0 × 1.0 × 1.0 mm^3^, acquisition time 6:42. Diffusion tensor imaging: single shot 2D SE EPI, TR 10,000 ms, TE 94 ms, diffusion values b = 0 s/mm^2^, b = 1000 s/mm^2^, diffusions directions 61, slice count 69, voxel size 2.0 × 2.0 × 2.0 mm^3^, acquisition time 11:40. Deformation correction of the EPI sequence according to Zaitsev et al. 2004 [26].

#### Fiber tracking

Deterministic FT was performed on a Linux workstation using StealthViz DTI (Medtronic Navigation, Louisville, CO, USA). An internal transfer procedure was used to fuse the line-graphic depiction of the DRT to the DICOM image that further serves for navigation purposes. With this procedure, the DRT becomes part of the stereotactic planning data. Our group has described before fiber tracking of the cerebello-thalamo-cortical network (DRT) and surrounding structures (CST, ml) [1–4].

### Surgical procedure

After administration of standard antibiotic prophylaxis, a stereotactic frame (Leksell, Elekta, Stockholm, Sweden) was placed under local anesthesia. A CT scan was performed and the image data were transferred to the planning workstation (Framelink 5.0, Medtronic SNT, Louisville, CO). The previously acquired MRI sequences and the DTI FT rendition of the DRT (as part of the stereotactic DICOM data) were co-registered with the stereotactic CT scan and the trajectories were planned taking into account MCP (mid-commissural point) coordinates (for STN we typically use: *x* = 12; *y* –2, *z* = –4) and imaging of the targeted structures (DRT and STN). Where necessary, based on the imaging, the target was refined based on the direct visualization of the structures.

**Fig. 2:**
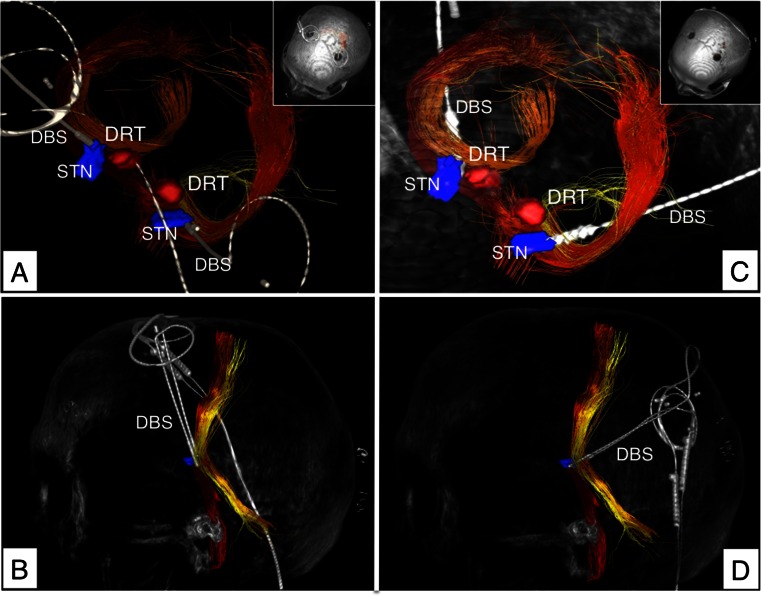
Case no. 1, three-dimensional rendition. *Left column* (**a**, **b**): Initial implantation attempt with traditional approach to the STN which did not sufficiently alleviate right upper extremity tremor. DBS electrode tip barely touches the DRT, bilaterally. *Right column* (**c**, **d**): Second implantation over the here proposed parietal approach. Note how one-pass DBS reaches the STN and the DRT, simultaneously. This second approach led to satisfactory tremor control. *DRT* dentato-rubro-thalamic tract, *STN* subthalamic nucleus (*blue*), *DBS* deep brain stimulation electrode (octopolar)

**Fig. 3:**
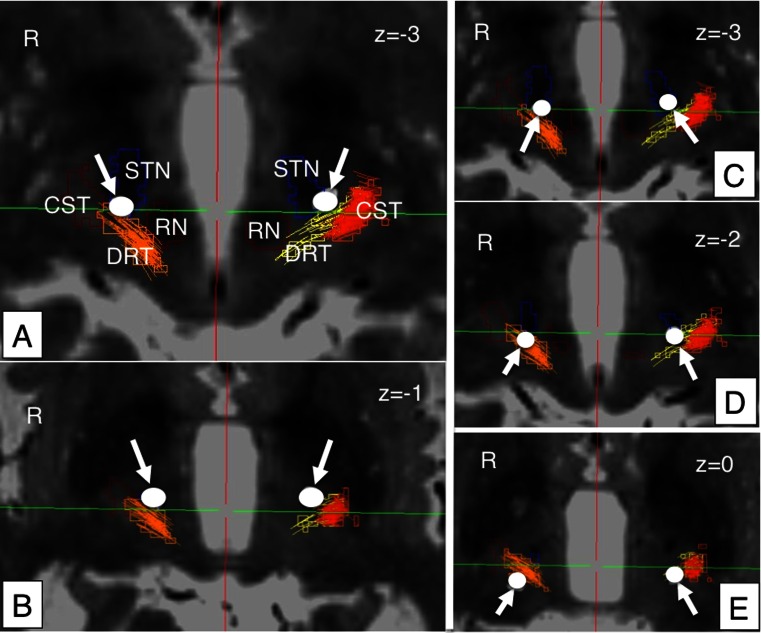
Case no. 1, continued. *Left column* (**a**, **b**): Initial implantation attempt without sufficient reduction of upper extremity tremor. DBS electrodes (*white arrows*) barely touch the DRT, bilaterally. *Right column* (**c**, **d**): Newly proposed parietal approach. DBS electrodes traverse the DRT, bilaterally. *ACPC* parallel axial views. *Z* indicates vertical coordinate. *Negative z* indicates millimeters below ACPC place. *DRT* dentato-rubro-thalamic tract, *CST* cortico-spinal tract, *RN* red nucleus, *STN* subthalamic nucleus (*blue*); *white arrows* (*circles*) indicate deep brain stimulation electrode contacts

**Fig. 4:**
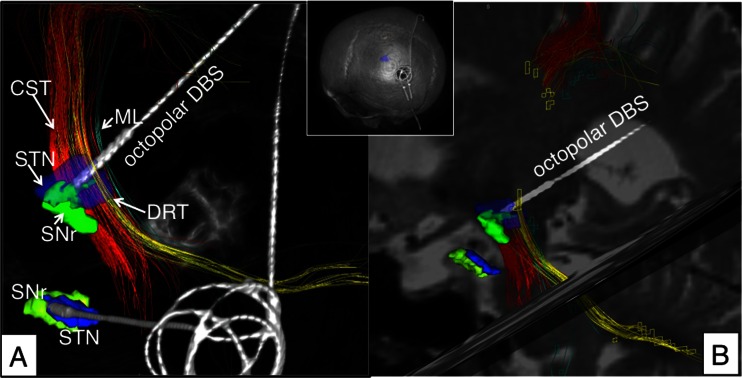
Case no. 2. **a** Three-dimensional rendition after one-pass implantation. DBS electrode reaches the STN with its tip after traversing the DRT. **b** Quasi-sagittal reconstruction along right electrode traversing the posterior horn of the lateral ventricle. *CST* cortico-spinal tract, *STN* subthalamic nucleus, *SNr* substantia nigra, *ml* medial lemniscus, *DRT* dentato-rubro-thalamic tract

The bilateral DBS electrode implantation was performed under local anesthesia with the patient in a semi-sitting position. Using a micro-targeting drive (MicroTargeting Star Drive M/E System, FHC Inc., Bowdoin, ME) a single test electrode (Cosman Medical, Inc., Burlington, MA, 1.3-mm diameter, 2-mm exposed tip) was inserted through a parietal burr hole in the cranium. Because of anticipated transventricular routes we elected not to use sharp microelectrodes for micro-recording (MER) but instead relied on the imaging taking into account that the anterior, lateral and superior STN (= sensorimotor or dorsolateral STN) must be targeted [6]. Macro-stimulation was performed to confirm a contralateral clinical benefit (tremor reduction at a low threshold for DRT, additional reduction of bradykinesia and rigidity more distally on the trajectory, in the STN) and to test for side effects (at a high threshold) in 2-mm steps starting 4 mm above the individual target regions. In the two cases presented here, no change of trajectories was necessary based on the intraoperative stimulation results. The definitive DBS electrodes (octopolar electrode, Boston Scientific, Natick, MA, USA) were then implanted under lateral X-ray control. An implantable pulse generator (Vercise™, Boston Scientific, Natick, MA) was implanted under general anesthesia in the sub-clavicular region during the same procedure. Postoperatively, the patients underwent helical 3D CT scans to corroborate the final DBS electrode locations (cf. Figs. 2, 3, and 4).

### Clinical evaluation

Clinical response of motor symptoms was evaluated using the Unified Parkinson’s Disease Rating Scale (UPDRS), part III (motor examinations). UPDRS scores were measured after combined STN and DRT stimulation in Med ON, Stim ON, and compared to preoperative Med ON. For tremor, the UPDRS items related to this symptom were calculated separately. In order to assess changes in medication, the l-dopa equivalent doses (LED) were calculated as suggested in the literature [24].

## Results

### Evaluation of tractography studies

The DRT could be reconstructed and visualized in every case as well as the corticospinal tracts (CST) and the medial lemnisci (ml). The fusion of the postoperative helical CT scans to the planning data in combination with tractography results (StealthViz DTI, Medtronic, USA) proved involvement of the DRT and the STN in both cases (cf. Figs. 2, 3, and 4). The anatomical sites with respect to effective contacts (EC) based on the DTI and CT superimposition were identified in both patients. Postoperatively (at day 3), a monopolar review was performed looking for the threshold for beneficial effects and side effects (typically capsular effects or paraesthesias from medial lemiscus). A clear distinction between ECs in the STN (reducing rigidity, bradykinesia, and tremor) and contacts located in the DRT (reducing tremor) could be made based on the stimulation results and were congruent with the imaging evaluation.

### Stimulation parameters

Detailed stimulation parameters are given in Table 2. Patient 1 reacted over time with some speech alterations (dysarthria; cerebellar, not capsular) elicited clinically from his left DRT program (cf. Table 2, EC5). A reduction of pulse width to 30 µs alleviated this immediately, something that is known from STN DBS [21]. After a brief period of dyskinesia (leg) in patient one, contacts were changed and a more proximal contact was stimulated for DRT (EC4 - > EC5). Because patient 2 reacted with a brief period of right lower extremity dyskinesias during activation of the tip of her left electrode (STN), we elected to switch this contact off. She is momentarily not stimulated in her left STN but obviously would react with anti-bradykinetic effects.

### Clinical results

Clinical results are summarized in Table 1. Patients tolerated surgery well and no worsening of the neurostatus, no infections, and no intracranial hemorrhages were seen. In both patients, DRT and STN were reached in one-pass bilaterally (cf. Figs. 2, 3, and 4). Combined DRT/STN-DBS reduced global UPDRS III scores as compared to preoperative medication ON condition. Furthermore, stimulation of both target regions at once was able to significantly alleviate tremor in both cases (tremor items of UPDRS reduced by 78 and 75 %, respectively). Patient 1 reported superior tremor control (especially over his right upper extremity) when compared to the situation under traditional STN DBS. Despite some dyskinesias (STN) in both patients during the titration phase, and some transient dysarthria in patient 1, no side effects were seen. We did especially not detect any clinical signs of neuropsychiatric effects although we did not specifically test for this. However, the patients’ spouses confirmed our clinical impression.

### Case reports

#### Case no. 1

This 75-year-old male suffers from EQT IPS diagnosed 12 years ago. In recent years, he experienced significant fluctuations of his therapy with an insufficient control of his right-sided resting and some postural tremor under medication (pramipexole, rasagilin, l-dopa). During neurological evaluation, no contraindication for DBS could be identified. He was classified as treatment-resistant. The l-dopa challenge test showed an improvement of 54 % on the UPDRS motor score (63 OFF, 29 ON). His tremor reacted somewhat less with only 12 % (16 OFF, 14 ON in the tremor items of UPDRS III). Despite this low response of his tremor to dopamine and because of his mild fluctuations with some dyskinesias over the days, he was selected for STN surgery, which at this moment appeared advisable. STN DBS was successfully performed in a standard anterior approach (cf. Figs. 2a, b and 3a, b). All symptoms improved; however he was not quite satisfied with the overall reduction of the right upper extremity tremor, which posed a problem under different stimulation patterns. This was despite a later proven relative proximity of the electrode to the DRT, bilaterally (cf. Figs. 2 and 3). Two months after implantation, an infection occurred and the complete system was removed. In light of the insufficient tremor control, he was offered the parietal approach in order to also improve this symptom. He remained under medication for 6 weeks and a new DBS system was implanted over the parietal fiber tractography approach (newly described here, Figs. 2c, d and 3c–e). After initiation of stimulation (cf. Table 2) and a titration phase, a much better symptom control was achieved (cf. Table 1) including satisfactory tremor control (global UPDRS III 62 % improvement, tremor items 78 % improvement) and improved sleeping. The patient showed a reduction in dopamine medication (LED: preop 665 mg, postop 200 mg; 70 % reduced). The follow-up time was 5 months.

**Table 1:** Patient characteristics

Patient no.	Gender/age	Dx	Preop. UPDRS III (med off)		Preop. UPDRS III (med on)		Improv. (%)		Postop. UPDRS III (med on/stim on)		Improv. (%)		Follow-up (months)
			gl	tr	gl	tr	gl	tr	gl	tr	gl	tr	
1	m / 75	TD IPS	63	16	29	14	54	12*	11	3	62	78	5
2	f / 76	TD IPS	56	11	41	8	26	27*	11	2	73	75	8

*Dx* diagnosis, *TD IPS* tremor-dominant idiopathic Parkinson syndrome, *UPDRS III* motor part of the Unified Parkinson’s Disease Rating Scale, *gl* global, *tr* tremor related, * lack of dopamine effect on tremor reduction

#### Case no. 2

This 76-year-old woman was diagnosed with EQT IPS 15 years ago. Over the years, the disease progressed into TD IPS with some mild fluctuations and a morning right lower extremity dystonia. The predominant symptom was right-sided (upper and lower extremity) resting tremor, although at the time of indication for surgery resting tremor at all extremities was present (5 Hz).

The l-dopa challenge test showed an improvement of globally 26 % (56 ON, 41 OFF) and only 12 % (12 ON; 8 OFF) for the tremor items of the UPDRS III. The patient was determined as treatment-resistant and surgery was offered. Surgery was performed uneventfully. Under stimulation, her symptoms markedly improved, and especially the tremor was almost immediately alleviated. UPDRS III was reduced to 11, globally (73 % improved) and 2, for the tremor items (75 % improved). A marked reduction of the medication followed during the titration phase of stimulation (cf. Table 2; LED: preop 716 mg; postop 250 mg; 65 % reduced). Follow-up time was 8 months.

**Table 2:** Stimulation parameters

Pat. no.	Side	STN	DRT
1	lt	EC2, (–), 1.0 mA, 60 µs	EC5, (–), 2.0 mA, 30 µs EC6, (+)
	rt	EC9, (–), 2.0 mA, 60 µs	EC10, (–), 1.5 mA, 60 µs
2	lt	–	EC4, (–), 1.8 mA, 60 µs EC5, (–), 1.2 mA, 60 µs
	rt	EC9, (–), 1.0 mA, 60 µs	EC10, (–), 1.85 mA, 60 µs EC11, (–), 1.85 mA, 60 µs

All stimulated with frequency of 130 Hz

*EC* effective contact (1–8 left, 1 most distal; 9–16 right, 9 most distal)

*rt* right, *lt* left, *STN* subthalamic nucleus, *DRT* dentato-rubro-thalamic tract

## Discussion

### Surgery of the STN utilizing a parietal approach

Anatomically, the Vim/DRT as the usual target region for tremor typically lies more superficially and posteriorly (with respect to the MCP reference system) than the STN target region [3] (cf. Fig. 1). Therefore, a parietal approach is required in order to allow traversing (and later modulating) both regions at the same time or differentially. In the typical approach to the STN region anteriorly, the antero-lateral and superior STN is targeted [6]. In this traditional approach, the Vim/DRT region is likely missed anteriorly, since it is located more superficial and posterior. If the traditional approach is used, this region can only occasionally be reached via an electric field that spreads in to this region posteriorly (cf. Fig. 3a, b). Typically, the target for tremor surgery (Vim/DRT) is approached by a separate trajectory anteriorly [3] that will not or only occasionally end in the posterior STN region (pSTR, cf. Fig. 1). However, our simulation studies showed that it could be safely reached on the way to the STN posteriorly. In order to achieve an optimal placement for tremor reduction, it appears that the STN region can be skewered posteriorly, while traversing the DRT in the thalamic region over this parietal route (cf. Figs. 1a; 2c, d; 3c–e). The posterior (parietal) approach can be used to reach the thalamic and the STN region at the same time over one single brain-perforating path. This approach has until today anecdotally been reported as a salvage strategy pathway to reach the STN if in the frontal region an infection has occurred (similar to our case 1) [27]. Our approach here is an evolution from this first description in a different patient group namely TD IPS. For targeting purposes, the STN is directly visualized on T2-weighted MRI sequences. We additionally utilize the DTI FT technology that we already use routinely in daily clinical practice to visualize the DRT [2–4, 7]. Planning this approach might be demanding, since a three-point trajectory (STN, DRT, entry point) has to be found that safely enters into the target region. However, two of the points are directly visualized with the MRI technology and there will typically be some room to move in the cortical entry zone. More medial approaches will penetrate the ventricles (like in case 2, here, cf. Fig. 4).

More lateral approaches might have the danger of damaging (sub-) cortical eloquent brain regions (Wernicke’s area on the left, etc.). However, there is no literature that supports a higher bleeding risk of this proposed posterior trajectory. From the regulatory point, it is left to the surgeon’s discretion how he approaches the STN. In our two cases, the parietal approach allowed a safe placement of electrodes in both the STN and the DRT.

### Trans-ventricular trajectories

Some authors discuss additional risks of traversing the ventricle during DBS placement. There is a possible risk of reduced placement accuracy and an increased bleeding risk form traversing ependymal surfaces. Kramer and coworkers recently showed equal accuracy and a similarly low bleeding risk when traversing the ventricle for subthalamic DBS including the use of MER [15]. In the SANTE trial on anterior nucleus DBS for partial epilepsy, only trans-ventricular approaches were used. No symptomatic bleedings (4.5 % incidental hemorrhages on imaging) were detected in 110 cases [10]. While we are aware of possible problems, we believe that with the methods applied (macroelectrode, 15-mm rigid tip of the DBS electrode for accurate placement while traversing the ependymal surfaces; high-resolution T1W MRI to plan a safe trajectory with respect to vascular anatomy) accurate and safe placement is possible.

### Intraoperative adjustment of trajectories based on stimulation results

In the cases described here, a change of the trajectory was not necessary. One could envision that an adjustment of the trajectory might be a difficult task. Certainly an adjustment will not be just a parallel tract. There are multiple restrictions from the anatomical and functional environment. However, stimulation of the DRT teaches us that the tract can be modulated at different parts of it and will still be effective [2, 3]. In this respect, the adjustment would look more like an idealized “ice cone” with a rather fixed tip in the anterior lateral and superior STN [6] and larger adjustment possibilities in the DRT and cortical portion of the trajectory.

### Risk of simultaneous stimulation

There are patients that have electrodes located in Forel’s field above the STN who benefit from electrodes located there with better tremor control than in traditional STN surgery (some researchers actually think that the benefit of STN-DBS stems from stimulation of the hyperdirect pathway (HDP) above the STN in Forel’s field). This occasionally happens, if the DRT region (because of anatomical variations) in the traditional anterior approach is close enough to the more proximal electrodes (Figs. 1b; 2a, b; 3a, b). In the approach chosen, we have operationalized this in two cases and ensured the accessibility of the DRT by choosing a parietal approach.

With this technique, patients with TD or EQT IPS can—depending on their age, and because of the higher incidence of neuropsychiatric side effects in pure STN DBS—receive STN DBS, DRT DBS, or gradually both. This possibly could have advantages during the course of the disease progression because of the adjustability of the therapy (STN for bradykinesia, rigidity, and tremor; DRT for tremor).

There are anecdotal reports of patients with pure thalamic DBS that later received STN-DBS in a second surgery [11, 19]. There is no indication that hints towards detrimental effects of simultaneous stimulation of both targets (DRT and STN) in these reports. The other is also known, patients with STN-DBS that later received thalamic DBS because of a later loss of tremor control over the course of the disease. According to our limited experience, the combined approach presumably would allow for a better symptom control in TD and EQT IPS.

Interestingly, we have observed a very low but nevertheless effective stimulation amplitude in both target regions. These low amplitudes correspond to a dramatic clinical improvement and a clear reduction of the LED. We are not quite sure what the reason is but it might be that modulating fiber structures from another angle than usually (in the anterior approach) might prove be superior.

### Limitations

We have extensively discussed the limitations of the fiber tractography approach to DBS (anatomic rendition of fibers with DTI, crossing and kissing fibers) and the limitations of the deterministic DTI technology in our previous publications [2–4, 7]. The deterministic approach is prone to a higher subjectivity than other approaches (e.g., probabilistic tractography). The extension of a fiber tract very much depends on the software used and on a variety of other factors (region of interest, algorithm, multiple tensor models, etc.) [1]. The “anatomical truth” of a fiber tract like the DRT depends on the definition of a variety of parameters and on the imaging data it originates from (e.g., most deterministic approaches will not show a pontine decussation of the DRT). Moreover, it is difficult to determine a “true accuracy” of the deterministic approach [5, 22]. In a recent study, we found a level of agreement to be ±2.5 mm when comparing electrophysiological distance measurements with DTI. In our experience, however, the accuracy of the method is higher [18]. There are examples in the literature for detrimental results of uncritical use of deterministic tractography in conjunction with neuro-navigation while ignoring electrophysiology [13]. However, when cautiously applying this technology, it can be safely used for navigation and targeting purposes [2, 3, 5, 7].

Certain limitations, primarily owing to the case study character of this report, have to be discussed: This report shows that a one-pass threading of two target regions (STN and DRT) is possible without obvious detrimental effects to the patients. Tremor could more optimally be investigated with a more detailed tremor rating (e.g., Fahn–Marsden Tremor rating scale) and with blinded raters using video rating. We perform this in our clinical study. We have shown that tremor and, to a certain extent other IPS symptoms, can be positively influenced with stimulation of these targets. We have, however, not differentially tested the effects of singular STN or DRT stimulation via this route. This certainly would be an interesting question. The focus in the treatment of the two patients was the alleviation of clinical symptoms of IPS. It would, however, have been interesting to see to what extent dopaminergic medication could be decreased depending on the target stimulated and if stimulation of the STN and DRT over the parietal approach could be considered in much younger patients with EQT IPS that might later develop strong fluctuations. We cannot address these questions with our results here. This and the question if a combined stimulation of both regions is superior to stimulation of single structures and the effect on the patients’ quality of life is the focus of a clinical study that we have just initiated and which is currently recruiting.

## Conclusions

The proposed parietal approach threading the STN and DRT in one pass appears to be feasible and safe. A differential stimulation of the two target regions (DRT and STN) can be performed with the implanted DBS system tailored to the patients’ symptoms, within typical stimulation ranges. Clinically, no neuropsychiatric effects were seen. Even with a trans-ventricular approach in one patient, electrodes could be safely and accurately placed in both target regions. We hypothesize that the combined stimulation of the STN and the DRT target could be superior with respect to the overall symptom reduction as compared to the singular stimulation of one individual target region (STN or DRT). In one patient who was previously stimulated in the STN (with a traditional anterior approach) this was certainly the case. In this respect, our pilot data might suggest a new and viable therapeutic option to treat the subgroup of TD and EQT IPS and with tremor as the predominant symptom. However, if the results could be replicated in a larger patient group, this work would have the chance to change standardized treatment with DBS for the whole patient group of TD or EQT IPS patients. A clinical study (recruiting) to further investigate this approach in a larger patient group (OPINION: www.clinicaltrials.gov; NCT02288468) is the focus of our ongoing research.

## Abbreviations

cZI: Caudal zona incerta
CT: Computed tomography
DBS: Deep brain stimulation
DRT: Dentato-rubro-thalamic tract
DTI: Diffusion tensor magnetic resonance imaging
EC: Effective contact
EQT: Equivalent type
FT: Fiber tractography
IPS: Idiopathic Parkinson syndrome
LED: l-dopa equivalent dose
MCP: Mid-commissural point
MRI: Magnetic resonance imaging
PD: Parkinson’s disease
pSTR: Posterior subthalamic region
TD: Tremor-dominant
Vim: Ventral intermediate nucleus of thalamus
